# Peritoneal implantation of pheochromocytoma – pheochromocytomatosis: a case report and mini review

**DOI:** 10.3389/fendo.2025.1679629

**Published:** 2025-11-03

**Authors:** Erik Bényei, András Laki, Gergely Kiss, Zsolt Varga, Miklós Tóth, Judit Tőke

**Affiliations:** ^1^ Faculty of Medicine, Department of Internal Medicine and Oncology, Semmelweis University, Budapest, Hungary; ^2^ Faculty of Medicine, Medical Imaging Centre, Semmelweis University, Budapest, Hungary; ^3^ Faculty of Medicine, Medical Imaging Centre, Department of Nuclear Imaging, Semmelweis University, Budapest, Hungary

**Keywords:** pheochromocytoma, paraganglioma, pheochromocytomatosis, recurrence, peritoneal implantation

## Abstract

**Introduction:**

Pheochromocytomatosis, defined as the implantation of pheochromocytoma cells to the intraoperatively opened surfaces during surgical manipulation, is an infrequent complication of surgical intervention of pheochromocytomas. Only a handful of pheochromocytomatosis cases have been reported since the first case was described in 2001.

**Case report:**

In 2011, a 33-year-old male patient presented with episodic palpitations and hypertensive surges triggered by physical activity. Imaging revealed a left adrenal tumor, which showed intense radiopharmaceutical uptake on ^131^I-metaiodobenzylguanidine ([^131^I]MIBG) scintigraphy. Urinary analysis of metanephrines confirmed pheochromocytoma, and laparoscopic left-sided adrenalectomy was performed. Owing to the large tumor size, intraoperative fragmentation was necessary for removal. The patient remained asymptomatic for five years. In 2016, recurrent paroxysmal symptoms prompted imaging, revealing a lesion at the left renal hilum. During the reoperation in 2017, multiple peritoneal tumor deposits were observed and later confirmed histologically. Over the following years, the patient received conservative, symptomatic treatment with tolerable paroxysmal symptoms. In 2023, worsening symptoms led to the decision to commence three cycles of ([^131^I]MIBG) therapy, followed by alleviation of symptoms, and a decrease in biochemical parameters.

**Discussion:**

An extensive literature search for publications from the past 25 years identified 22 pheochromocytomatosis cases whose details were also summarized and analyzed. This condition appears to have a longer recurrence-free survival compared to patients’ cohorts with metastatic pheochromocytomas. Pheochromocytomatosis is usually characterized by a prolonged asymptomatic postsurgical interval, emphasizing the need for long-term follow-up with close biochemical and radiological surveillance. Treatment strategies parallel those used for advanced/metastatic pheochromocytomas.

## 1 Introduction

Pheochromocytomas and paragangliomas are catecholamine-producing tumors developing from the enterochromaffin cells of the adrenal medulla and the sympathetic ganglia. These tumors are characterized by the paroxysmal symptoms caused by these catecholamines, such as palpitations, sweating and hypertensive surges. The first-line treatment is surgical removal, which can provide a curative solution in cases with localized disease. Since all pheochromocytomas have metastatic potential, the term “malignant” is no longer used; instead, metastatic pheochromocytoma is applied when enterochromaffin tissue appears extra-adrenally at the time of diagnosis or during follow-up (1). Pheochromocytomatosis – defined as multifocal nodular implantation of the pheochromocytoma cells to the intraoperatively opened surfaces without the signs of distant metastases – is a rare, iatrogenic event caused by mechanical damage to the tumor capsule during surgery. This phenomenon was first defined in 2001 (2), and 22 cases have been reported in the literature since then.

In the past 25 years, we have treated and followed over 200 patients with pheochromocytomas at our endocrine referral center. Here, we report a patient’s history with pheochromocytomatosis who presented us with a therapeutic challenge during long-term management.

## 2 Case report

**Figure 1:**
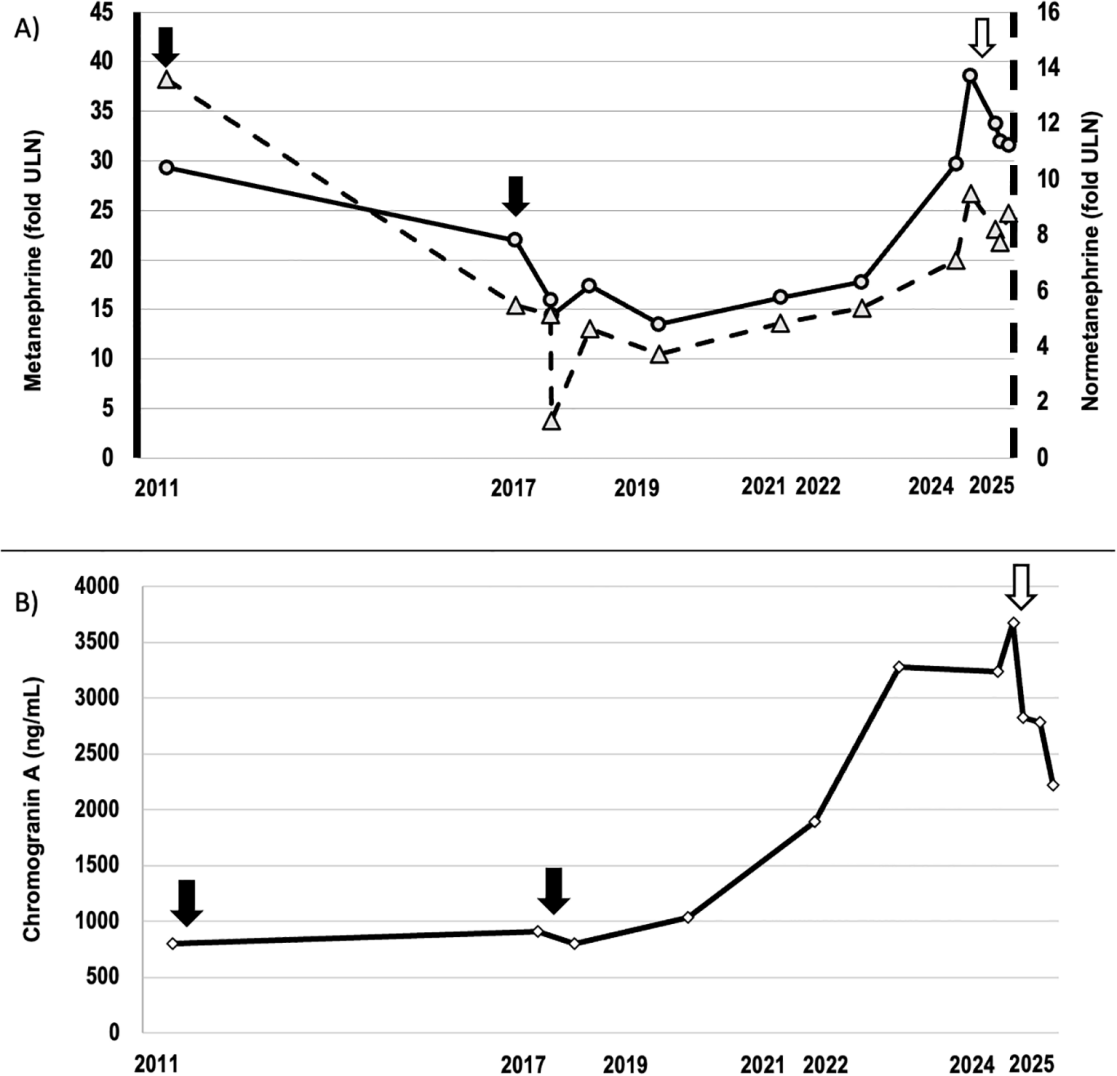
**(A)** Urinary metanephrine and normetanephrine excretion over time. **(B)** Serum chromogranin A concentrations between 2011 and 2024. Black arrows indicate surgical interventions, and white arrows mark the three cycles of ([^131^I]MIBG) therapy.

A 33-year-old male patient presented with episodic palpitations and hypertensive surges triggered by physical activity in 2011. During the diagnostic work-up, an abdominal MRI revealed a 4 x 4,8 x 6 cm tumor in the left adrenal gland, which exhibited significant radiopharmaceutical uptake on ^131^I-metaiodobenzylguanidine ([^131^I]MIBG) scintigraphy. Urinary analysis showed elevated 24-hour metanephrine levels at 8860 µg/24h (normal range: 64 – 302 µg/24h) and normetanephrine levels at 7164 µg/24h (normal range: 162 – 527 µg/24h), alongside a serum chromogranin A level of 800 ng/mL (normal range: 19,4 – 98,1 ng/mL). The timeline of biochemical markers is shown in **Figure 1**. Based on these results, pheochromocytoma was diagnosed, and laparoscopic left adrenalectomy was performed in 2011. According to the surgical report, the large tumor could not be placed into the endobag; removal required deliberate fragmentation and manual extraction through an enlarged port. Histological investigation confirmed the diagnosis of pheochromocytoma. For the first five years after surgery, the patient remained asymptomatic and radiological follow-up showed no signs of recurrence.

**Figure 2:**
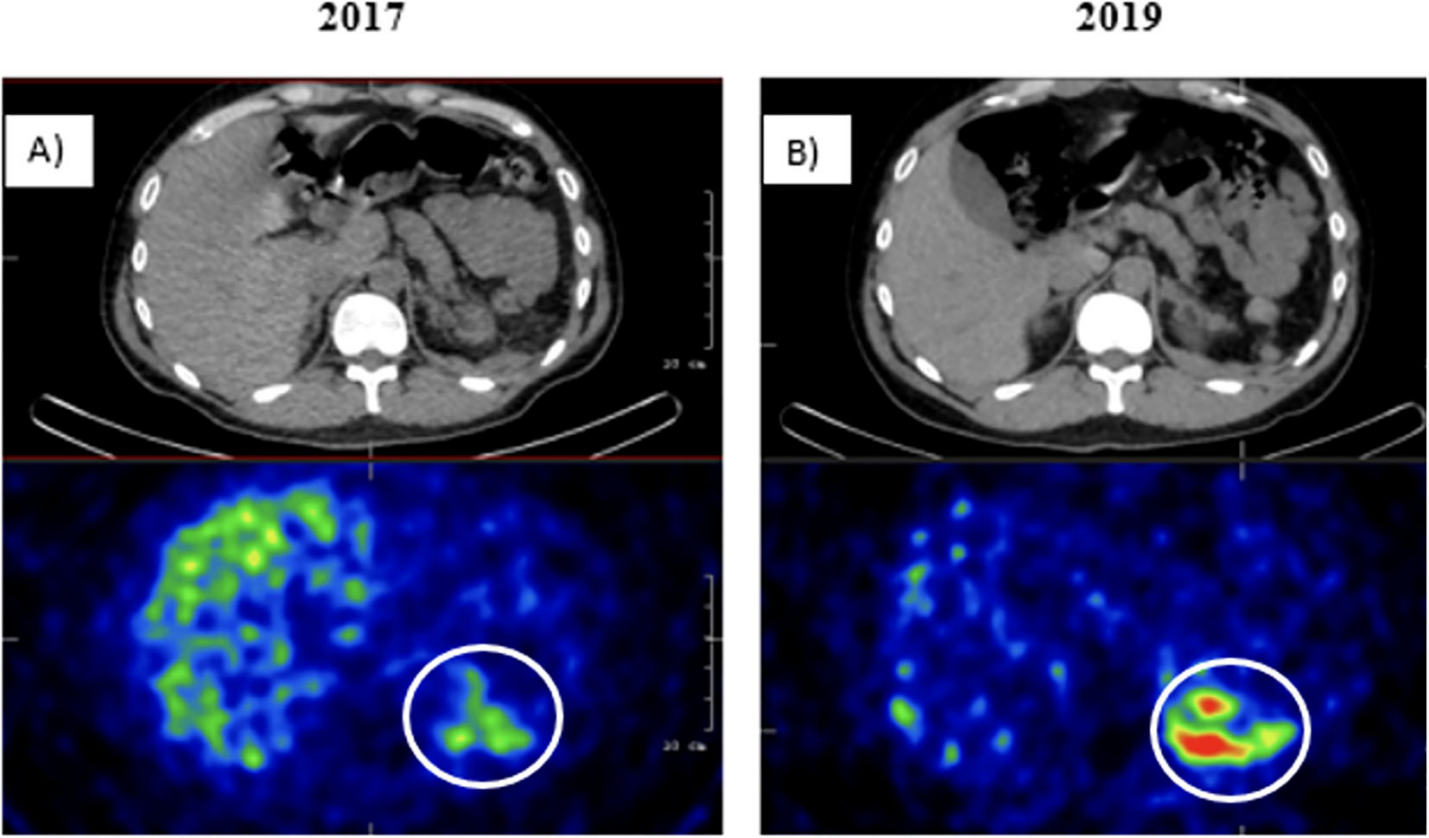
([^131^I]MIBG) scintigraphy SPECT/CT images in **(A)** 2017 and **(B)** 2019 show multiple peritoneal lesions with significant radiopharmaceutical uptake in the left hypochondrium. No distant metastases were detected.

In 2016, the patient presented with recurrent paroxysmal episodes with palpitations and hypertensive surges. Abdominal MRI and ([^131^I]MIBG) scintigraphy revealed a lesion at the left renal hilum, consistent with a tumor recurrence *(* **Figure 2** *)*, which was further verified by the elevated urinary excretions of metanephrine (6641 ug/24h), normetanephrine (2882 µg/24h) and serum chromogranin A (910 ng/mL) levels *(* **Figure 1** *)*. During reoperation in June 2017, a massive perisplenic invasion was observed. Cytoreductive surgery was performed, including splenectomy and distal pancreatic resection. The surgeon noted multiple 2–3 mm tumor deposits forming only a partially resectable tumor-like mass in the left hypochondrium. Histological analysis confirmed peritoneal and retroperitoneal pheochromocytoma deposits. Postoperative ([^131^I]MIBG) scintigraphy indicated persisting multifocal peritoneal foci. Next-generation sequencing (ENDOGEN panel, Illumina MiSeq device) of DNA prepared from peripheral blood leukocytes revealed no pathogenic mutations in genes associated with hereditary pheochromocytoma/paraganglioma syndromes *(SDHA, SDHB, SDHC, KIF1B, EGLN1, FH, SDHAF2, MAX, SDHD, RET exon 10,11, VHL, TMEM127)*.

Over the following years, the patient received symptomatic drug treatment using alpha- and beta-blockers with tolerable paroxysmal symptoms. Regular radiological follow-ups revealed no new lesions compared to the imaging done in 2017; however, mild growth of previously described deposits was noted. Somatostatin-receptor scintigraphy turned out to be negative. In 2023, the patient’s symptoms became more frequent and intense, accompanied by radiological and biochemical progression *(* **Figure 1** *)*. In 2024 and 2025, three cycles of ([^131^I]MIBG) therapies were administered (3579 MBq, 3468 MBq and 3326 MBq). Post-treatment imaging showed a mild increase in lesions’ size and number. However, urinary metanephrine and normetanephrine excretions as well as serum chromogranin A concentration exhibited an unambiguous decrease following treatments, and the patient reported an alleviation of symptoms.

## 3 Literature review

A literature search was conducted using MEDLINE, EMBASE, and Web of Science databases. We identified 11 full-text publications including 22 patients with pheochromocytomatosis reported between January 2000 and December 2024 (2–12). Although a few case reports describing pheochromocytomatosis date back nearly fifty years, these were excluded due to limited data quantity and/or quality and the substantial evolution in diagnostic and therapeutic procedures over time (13, 14). Publications were identified through keyword searches using the terms “pheochromocytomatosis”, {[“pheochromocytoma” OR “paraganglioma”] AND “recurrence”}, as well as by citation chasing. Only English-language publications with full-text availability were considered, and inclusion was based on a detailed full-text evaluation.

The clinicopathological characteristics of these 22 patients completed with our presented case are summarized in **Table 1**, while treatment and follow-up details are presented in **Table 2**. The mean age at the time of initial diagnosis was 42 ± 13.7 years. Pheochomocytomatosis was diagnosed predominantly in females (60.9%). Mean primary tumor size was 6.0 ± 2.2 cm and the majority of adrenalectomies (69.6%) was performed laparoscopically. Three patients were reported to have MEN2A syndrome, and one to have neurofibromatosis type 1. The surgical or the pathological reports typically described inadvertent tumor fragmentation or capsule rupture. All publications reported patients to be tumor-free after initial surgery, confirmed with resolution of clinical symptoms and normalization of biochemical parameters. Median (minimum – maximum) recurrence-free survival across all cases was 72 (23 – 180) months. Diagnostic workup for recurrence, prompted by positive biochemical follow-up, was conducted in only 5 patients (21.7% of cases), whereas in 12 patients (52.2% of cases) pheochromocytomatosis diagnoses were initiated due to recurrent symptoms. Most frequently performed imaging procedures were CT and ([^131^I]MIBG) scintigraphy, followed by abdominal MR scans. All patients underwent a second surgical intervention. At least 13 (56.5%) of them required further surgical interventions or additional therapies. The postoperative follow-up duration was highly variable. At the time of publication, 6 (26.1%) patients were reported to be in remission, 8 (34.7%) to have stable disease, and 2 (8.7%) died from tumor progression. Notably, out of the 23 patients, only 2 (8.7%) were reported to be tumor-free at least one year following the second surgery (**Table 2**).

**Table 1:** Clinicopathological characteristics at the time of initial surgery for all patients reported with pheochromocytomatosis.

Publication	No of cases reported	Sex	Age	Primary tumor size (cm)	Side	Surgery type	Tumor fragmentation mentioned in the surgical and/or pathological report	Tumor-syndrome/genetics
Li, 2001 (2)	3	m	29	5.5	l	laparoscopy	"Tumor break and spillage"	sporadic (based on phenotype)
		f	47	5.7	r	laparoscopy	"Friable tumor"	sporadic (based on phenotype)
		f	47	6.5	l	laparoscopy	"Extensive manipulation of the ill-defined adrenal tumor"	sporadic (based on phenotype)
Robledo, 2010 (3)	2	f	37	10.0	r	laparotomy	"Difficult resection because of size and vascularity"	*no data*
		f	35	*nd*	bilateral	*nd*	*nd*	MEN2A (based on phenotype)
Rafat, 2014 (4)	5	f	45	7.0	l	laparoscopy	"Capsule rupture"	NF1 (based on phenotype)
		f	63	7.0	r	laparoscopy	"Capsule rupture"	hypermethylated phenotype FH (fumarate hydratase) mutation
		f	54	11.0	r	laparoscopy	"Deliberate tumor fragmentation"	*no data*
		m	63	2.5	l	laparoscopy	"Inadvertent tumor fragmentation"	*no data*
		m	39	4.0	r	laparotomy	No fragmentation reported	*no data*
Pogorzelsi, 2015 (5)	1	f	29	6.0	r	laparotomy	"Capsule rupture"	sporadic (based on genetic analysis)
Tramunt, 2016 (6)	1	m	33	6.5	l	laparoscopy	R0 resection	MEN2A (RET p.Cys634Arg)
Javid, 2017 (7)	1	f	42	4.6	l	laparotomy	"Tumor disruption"	sporadic (based on phenotype)
Yu, 2017 (8)	1	f	64	6.0	r	laparoscopy	"Capsule rupture"	TMEM127 c.570del
Weber, 2019 (9)	5	m	33	10.0	l	laparotomy	No fragmentation reported	sporadic (based on genetic analysis)
		f	53	7.0	r	laparoscopy	No fragmentation reported	sporadic (based on genetic analysis)
		m	49	*nd*	l	laparotomy	No fragmentation reported	sporadic (based on genetic analysis)
		f	19	5.0	l	laparoscopy	No fragmentation reported	sporadic (based on genetic analysis)
		f	40	5.3	l	laparoscopy	No fragmentation reported	MEN2A
Ferrer-Inaebnit, 2021 (10)	1	m	13	4.0	r	laparoscopy	"Non-assessable margins"	*no data*
Auerbach, 2022 (11)	1	m	38	4.8	l	laparoscopy	"Capsule rupture"	sporadic (based on genetic analysis)
Green, 2022 (12)	1	f	60	2.5	l	laparoscopy	"Capsule rupture"	GLCCI1-BRAF fusion
Bényei, 2025	1	m	33	6.0	l	laparoscopy	„Deliberate tumor fragmentation during surgery”	sporadic (based on genetic analysis)

**Table 2:** Tumor recurrence and follow-up for all patients reported with pheochromocytomatosis.

Publication	No of cases reported	Time to recurrence (months)	Reason for diagnostic workup of recurrence	Radiological findings		Visceral metastasis during follow-up?	Treatment			Disease state at the time of publication	Tumor-free at least one year after reoperation?
				CT	([^131^I]MIBG) - scintigraphy		Reoperation	([^131^I]MIBG) therapy	Other therapies		
Li, 2001 (2)	3	34	recurrent symptoms	–	+	–	1x	–	–	*no data*	*no data*
		48	recurrent symptoms	+	+	–	1x	–	–	*no data*	*no data*
		46	recurrent symptoms	–	+	–	1x	–	–	*no data*	*no data*
Robledo, 2010 (3)	2	156	recurrent symptoms and elevated biochemical markers	–	+	–	1x	–	–	*no data*	*no data*
		86	elevated biochemical markers	*no data*	+	–	1x	–	–	*no data*	*no data*
Rafat, 2014 (4)	5	95	recurrent symptoms and elevated biochemical markers	+	+	liver	2x	3x	–	Stable disease	*no data*
		72	recurrent symptoms	+	+	–	1x	1x	–	Died from tumor progression	no
		42	recurrent symptoms and elevated biochemical markers	+	+	liver, lung and bone	3x	3x	–	Died from tumor progression	no
		24	recurrent symptoms	+	–	kidney	1x	–	interferonA, sunitinib	*no data*	no
		106	recurrent symptoms	*no data*	*no data*	–	1x	–	–	In remission	yes
Pogorzelsi, 2015 (5)	1	70	recurrent symptoms	+	+	–	1x	–	–	*no data*	*no data*
Tramunt, 2016 (6)	1	88	recurrent symptoms and elevated biochemical markers	+	+	–	2x	3x	–	In remission	no
Javid, 2017 (7)	1	180	recurrent symptoms	+	+	–	–	–	1x (radio-guided)	In remission	*no data*
Yu, 2017 (8)	1	72	recurrent symptoms	+	+	–	1x	–	–	In remission	*no data*
Weber, 2019 (9)	5	154	*no data*	*no data*	*no data*	–	1x	?x	–	Stable disease	no
		41	*no data*	*no data*	*no data*	–	1x	–	PRRT	Stable disease	no
		100	*no data*	*no data*	*no data*	–	1x	?x	–	Stable disease	no
		23	*no data*	*no data*	*no data*	–	1x	?x	–	Stable disease	no
		25	*no data*	*no data*	*no data*	–	1x	?x	–	Stable disease	no
Ferrer-Inaebnit, 2021 (10)	1	108	recurrent symptoms	*no data*	*no data*	–	2x	–	–	In remission	no
Auerbach, 2022 (11)	1	48	*no data*	*no data*	*no data*	–	1x	–	PRRT	Stable disease	no
Green, 2022 (12)	1	120	recurrent symptoms	+	+	–	1x	–	–	In remission	yes
Bényei, 2025	1	55	recurrent symptoms	–	+	–	1x	3x	–	Stable disease	no

## 4 Discussion

In addition to local recurrence and distant metastases, characteristic of malignant pheochromocytomas, pheochromocytomatosis represents another, infrequently reported type of tumor progression, which does not indicate malignancy. The removal of pheochromocytomas always poses a surgical challenge due to the tumor’s fragility and frequently soft consistency, with a rare complication being peritoneal tumor cell dissemination following damage to the tumor capsule (2). To characterize this condition better, we performed an extensive literature search.

Recurrence of pheochromocytoma after surgical removal is not considered rare, occurring in approximately 6.5 – 16.5% of cases, depending on the length of follow-up. It may be significantly more common in the presence of certain specific mutations (1, 15). Recurrence-free survival in pheochromocytoma patients is generally reported to be between 30 and 50 months (15–18), which is considerably shorter than the 72 months in pheochromocytomatosis patients of our meta-analysis. Pheochromocytomatosis case reports often describe a long, latent period – typically several years – following the initial surgery, during which patients appear biochemically and radiologically tumor-free. In most cases, the diagnostic investigation for pheochromocytomatosis was initiated after the recurrence of symptoms, despite documented tumor capsule rupture in nearly all cases. This highlights the critical importance of meticulous follow-up for these patients, encompassing regular radiological and biochemical evaluations while closely monitoring paroxysmal symptoms characteristic of pheochromocytomas. Due to the rarity and uncertain incidence of pheochromocytomatosis, robust survival data are lacking; however, one study reported better overall survival for patients with pheochromocytomatosis compared to those with metastatic pheochromocytomas (9).

Therapeutic approaches of pheochromocytomatosis largely corresponds to those used for metastatic pheochromocytomas. A „watch and wait” approach may spare patients the risks and side effects of other therapies in asymptomatic and radiologically stable disease cases. Cytoreductive (debulking) surgery can alleviate symptoms by reducing tumor burden and catecholamine excess. However, as repeated surgery led to remission in only a few cases, surgical interventions alone are unlikely to eliminate the long-term need for additional therapies. For tumors with sufficient radiopharmaceutical uptake, peptide receptor radionuclide therapy using radiolabeled somatostatin analogue or ([^131^I]MIBG) treatment may result in disease stabilization. Like in advanced pheochromocytoma management, somatostatin analogue therapy may also be a treatment option for tumors expressing somatostatin receptors. The efficacy of tyrosine kinase inhibitor sunitib, already approved for treating neuroendocrine tumors, has also been confirmed in patients with metastatic pheochromocytoma (FIRSTMAPP study) (19). Although Rafat et al. reported the inclusion of a patient with pheochromocytomatosis in this trial, the efficacy of sunitinib treatment remains unclear (4).

In recent years, two studies have examined somatic mutations of tumor cells in patients with pheochromocytomatosis. For the gene *TMEM127*, previously linked to pheochromocytomas (20), a new, likely pathogenic mutation (*c. 570delC*) was identified. In 2022, Green et al. proposed targeted systematic therapy with MEK and/or BRAF inhibitors following the identification of a *GLCCI1-BRAF* fusion gene (12). Identifying therapeutic targets could provide additional treatment options for therapy-resistant tumors.

A key strength of our case report lies in its detailed presentation of a rare and poorly understood condition, supported by comprehensive radiological and biochemical data. Another notable strength is the careful contextualization achieved by analyzing of similar cases reported in the literature. A limitation of our case report is the incomplete documentation of certain clinical details from the earlier years of follow-up. Regarding the literature review, a significant limitation is the heterogeneity in the pheochromocytomatosis management and follow-up across studies, which restricts the strength of conclusions drawn. Furthermore, the previously published cases span over two and a half decades, during which the clinical management of pheochromocytoma has undergone substantial changes, further limiting direct comparisons.

In conclusion, pheochromocytomatosis is an infrequent complication of pheochromocytoma surgery. Cautious intraabdominal handling of the tumor is key to preventing this adverse event. It is recommended that the surgery be performed by an experienced surgeon in a center specializing in adrenal surgery. In case of capsule rupture, rigorous radiological and biochemical follow-up is critical for the timely diagnosis and treatment of peritoneal dissemination, which may arise even several years after adrenalectomy. Analogously to the treatment of advanced, metastatic pheochromocytomas, therapeutic options to achieve stable disease include tumor debulking surgery, PRRT, somatostatin analogues and targeted systemic therapies. Adjuvant treatments are necessary to achieve stable disease.
